# *Escherichia coli* DNA polymerase III is responsible for the high level of spontaneous mutations in *mutT* strains

**DOI:** 10.1111/mmi.12061

**Published:** 2012-11-01

**Authors:** Masami Yamada, Masatomi Shimizu, Atsushi Katafuchi, Petr Grúz, Shingo Fujii, Yukio Usui, Robert P Fuchs, Takehiko Nohmi

**Affiliations:** 1 Division of Genetics and Mutagenesis, National Institute of Health SciencesTokyo, 158-8501, Japan; 2 Division of Medical Nutrition, Tokyo Health Care UniversityTokyo, 154-8568, Japan; 3 Unité Mixte de Recherche 7258, CRCM, Genome Instability and Carcinogenesis, Centre National de la Recherche ScientifiqueMarseille, F-13009, France

## Abstract

Reactive oxygen species induce oxidative damage in DNA precursors, i.e. dNTPs, leading to point mutations upon incorporation. *Escherichia coli* *mutT* strains, deficient in the activity hydrolysing 8-oxo-7,8-dihydro-2′-deoxyguanosine 5′-triphosphate (8-oxo-dGTP), display more than a 100-fold higher spontaneous mutation frequency over the wild-type strain. 8-oxo-dGTP induces A to C transversions when misincorporated opposite template A. Here, we report that DNA pol III incorporates 8-oxo-dGTP ≍ 20 times more efficiently opposite template A compared with template C. Single, double or triple deletions of pol I, pol II, pol IV or pol V had modest effects on the *mutT* mutator phenotype. Only the deletion of all four polymerases led to a 70% reduction of the mutator phenotype. While pol III may account for nearly all 8-oxo-dGTP incorporation opposite template A, it only extends ≍ 30% of them, the remaining 70% being extended by the combined action of pol I, pol II, pol IV or pol V. The unique property of pol III, a C-family DNA polymerase present only in eubacteria, to preferentially incorporate 8-oxo-dGTP opposite template A during replication might explain the high spontaneous mutation frequency in *E. coli* *mutT* compared with the mammalian counterparts lacking the 8-oxo-dGTP hydrolysing activities.

## Introduction

Excess oxidation is a major threat to the genomic integrity of most living organisms. Reactive oxygen species (ROS) are produced by normal cellular respiration, cellular injury or by exposure to environmental carcinogens and radiation. ROS generate a variety of altered purines and pyrimidines in DNA (Bjelland and Seeberg, 2003; Kamiya, 2003), thereby playing important roles in mutagenesis, carcinogenesis and ageing (Ames, 1983; Jackson and Loeb, 2001). It should be emphasized, however, that oxidized bases in DNA are introduced not only by direct oxidation of DNA but also by incorporation of oxidized deoxynucleoside triphosphates (dNTPs) into DNA by DNA polymerases (pols) (Michaels and Miller, 1992; Sekiguchi and Tsuzuki, 2002; Nakabeppu *et al*., 2006; Katafuchi and Nohmi, 2010).

7,8-Dihydro-8-oxo-dGTP (8-oxo-dGTP), a major form of oxidized dGTP in the cellular nucleotide pool, is a mutagenic substrate for DNA synthesis and the incorporation results in A:T to C:G mutations (Treffers *et al*., 1954; Yanofsky *et al*., 1966; Akiyama *et al*., 1989; Tajiri *et al*., 1995). When incorporated opposite A in the template DNA, 8-oxo-G can pair with incoming dCMP in the next round of DNA replication, then causing A:T to C:G mutations (Michaels and Miller, 1992; Kasai, 2002). To counteract the mutagenic 8-oxo-dGTP, *Escherichia coli* possesses a sanitizing enzyme, i.e. MutT, to hydrolyse 8-oxo-dGTP to the monophosphate form. When the *mutT* gene is inactivated, the mutation frequency of A:T to C:G transversions increases more than a 100-fold over the wild-type level (Yanofsky *et al*., 1966; Maki and Sekiguchi, 1992; Fowler *et al*., 2003). The high spontaneous A:T to C:G mutations in the *mutT* strain are almost completely suppressed when the *mutT* cells are cultured in anaerobic conditions, indicating the essential role of oxygen in the mutagenesis (Fowler *et al*., 1994; Sakai *et al*., 2006; Setoyama *et al*., 2011).

In mammals including humans, enzymes that possess similar activities to *E. coli* MutT are identified (Mo *et al*., 1992). Expression of human *MTH1* (*mutT* homologue-1) cDNA in *E. coli mutT* strain significantly suppresses the frequency of spontaneous mutations (Sakumi *et al*., 1993; Furuichi *et al*., 1994). Suppressive effects are also observed when mouse or rat cDNA is expressed in the *mutT* cells (Cai *et al*., 1995; Kakuma *et al*., 1995). These observations imply that the mammalian proteins may sanitize the nucleotide pools in the organisms, thereby reducing the spontaneous mutagenesis and carcinogenesis. In fact, deletion of the *Mth1* gene results in high frequency of tumour formation in several organs of mice (Tsuzuki *et al*., 2001). However, deletion of the mouse *Mth1* gene enhances the spontaneous mutation frequency only twofold, which is a striking difference from the strong mutator effects of *E. coli mutT* (Egashira *et al*., 2002). Although mammalian cells possess more than one enzyme to sanitize oxidized nucleotides (Bessman *et al*., 1996; Cai *et al*., 2003; Ishibashi *et al*., 2003), there may be other reasons to account for the marked difference in spontaneous mutagenesis between *E. coli mutT* and the mammalian counterparts.

In this study, we explored the potential involvement of the different *E. coli* DNA polymerases in the *mutT* mutator phenotype. *E. coli* possesses five pols, i.e. pol I (A family), pol II (B family), pol III (C family), pol IV (Y family) and pol V (Y family) (Nohmi, 2006), and pol III holoenzyme (pol III HE) is mainly responsible for the chromosome replication (McHenry, 2011). Pol III HE is a large dimeric complex, which is composed of pol III core complex including ε proofreading subunit, sliding clamp (β subunit) and the clamp loader (McHenry, 2011). Although A-, B- and Y-family pols are present in mammals, C-family pols are present only in eubacteria (Ito and Braithwaite, 1991; Filee *et al*., 2002). We disrupted the genes encoding pols I, II, IV and V, and characterized their mutator phenotypes. We also examined the biochemical properties of pol III*, the HE without β subunit (McHenry, 1988), incorporating 8-oxo-dGTP into DNA and effects of addition of β subunit on extension reaction upon incorporation of 8-oxo-dGTP by pol III* *in vitro*. Our results indicate that pol III HE may be responsible for the misincorporation of 8-oxo-dGTP into DNA and suggest that the erroneous nature of pol III HE uniquely present in bacteria might account for the strong mutator effects of *mutT* in *E. coli*.

## Results

### Deletion of the genes encoding pols in a *mutT* background

**Fig. 1:**
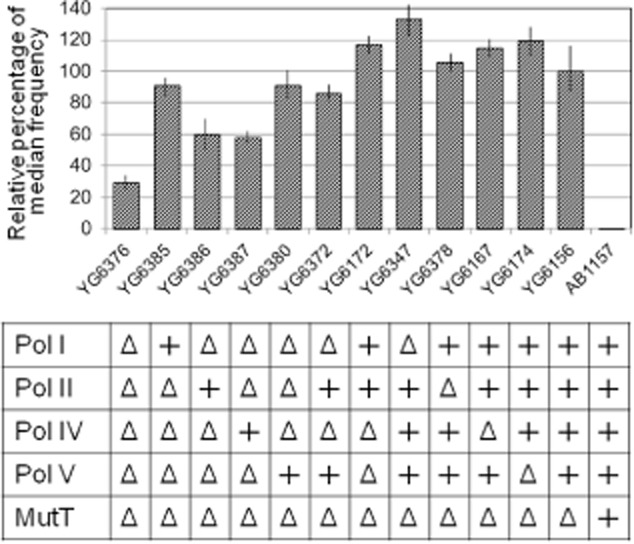
Mutation frequency of *mutT* strains with deficiency of DNA polymerase(s). Relative values (percentage) and the standard deviations of median frequency of rifampicin resistance mutations of *mutT* derivatives of *E. coli* are presented. Mutation assays were conducted at 30°C for strains with ΔKF and at 37°C for the other strains. The frequencies of strain YG6156 (Δ*mutT*) at 30°C and 37°C were set as 100%. The average mutation frequencies of YG6156 were 184 ± 58 × 10^−8^ at 30°C (*n* = 12) and 185 ± 57 × 10^−8^ at 37°C (*n* = 9). The mutation frequency of AB1157 at 37°C (the wild-type strain) was 1.3 ± 0.3 × 10^−8^ (*n* = 3). *n* represents number of repeated experiments. Mutation frequencies of other strains are presented in Table S1. The table under the graph indicates which polymerases are deficient (Δ) and proficient (+) in each strain.

To examine what pols are involved in the high spontaneous mutations in a *mutT* background, we deleted the genes encoding pol I (*polA*), pol II (*polB*), pol IV (*dinB*) or pol V (*umuDC*) in a *mutT* background. Deletion of *mutT* increased median frequency of rifampicin-resistant mutations more than 100 times without any damaging treatments to DNA (Fig. 1). None of single deletions of the *pol* genes reduced the median frequency of the *mutT*-deficient strain. Since pols IV and V preferentially incorporate 8-oxo-dGTP opposite template A *in vitro* (Yamada *et al*., 2006), we deleted both the *dinB* and *umuDC* genes and examined the spontaneous mutation frequency. However, the deletion of genes encoding two Y-family pols did not decrease the median frequency. We also deleted both the *polA* and *dinB* genes and examined the mutation frequency. Although pol I Klenow fragment (KF) and pol IV possess ability to extend primer DNA having 8-oxo-dGMP at the termini *in vitro* (see below and Fig. S3), deletion of the *polA* and *dinB* genes did not reduce the mutator phenotype substantially. Interestingly, when we deleted *polA*, *umuDC* and either *polB* or *dinB*, the spontaneous mutation frequency decreased by 40%. When we deleted all four pol genes, i.e. *polA*, *polB*, *dinB* and *umuDC*, the mutation frequency decreased by 70%. It should be emphasized, however, that the resulting penta mutant YG6376, i.e. Δ*mutT*, Δ*polA*, Δ*polB*, Δ*dinB* and Δ*umuDC*, still manifested more than 50 times higher spontaneous mutation frequency than the wild-type strain AB1157 (74 ± 20 × 10^−8^, YG6376 versus 1.3 ± 0.3 × 10^−8^, AB1157). These results suggest that pol III plays an important role in high spontaneous mutations in the *mutT* background, and also that other pols might have additional and redundant roles in the mutagenesis.

### Incorporation of 8-oxo-dGTP into DNA by pol III* *in vitro*

**Fig. 2:**
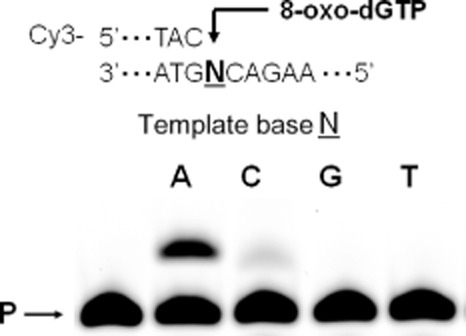
Incorporation of 8-oxo-dGTP by pol III*. The Cy3-labelled 18-mer primer/36-mer template (sequences 1, 0.1 μM) was treated with pol III* (1 nM) in the presence of 100 μM 8-oxo-dGTP. The reaction mixtures were incubated at room temperature for 1 min. The samples were analysed by denaturing polyacrylamide gel electrophoresis and visualized by the Molecular Imager as described in *Experimental procedures*. The alphabets shown in the figure indicate as follows: N, template base; A, adenine; C, cytosine; G, guanine; T, thymine; P, primer.

Next, we examined the specificity of pol III* incorporating 8-oxo-dGTP into DNA *in vitro* (Fig. 2). Pol III* preferentially incorporated 8-oxo-dGTP opposite template A in DNA. To examine the specificity quantitatively, we conducted kinetic analyses incorporating 8-oxo-dGTP into DNA (Fig. S1). Pol III* incorporated 8-oxo-dGTP opposite template A at concentration range of 8-oxo-dGTP from 0.05 to 10 μM (Fig. S1A). In contrast, pol III* incorporated 8-oxo-dGTP opposite template C at higher concentrations of 8-oxo-dGTP, i.e. 10 to 500 μM (Fig. S1B). The *f*_inc_ values for incorporation, i.e. the ratio between efficiency (*V*_max_/*K*_m_) of incorporation of 8-oxo-dGTP and that of incorporation of normal dNTP, were 5.6 × 10^−2^ and 2.9 × 10^−3^, respectively, opposite template A and template C (Table 1). It indicates that pol III* incorporates 8-oxo-dGTP opposite template A about 20 times more efficiently than opposite template C. It is remarkable that the apparent *K*_m_ value for incorporation of 8-oxo-dGTP opposite template A was 2.5 μM, which is similar to the values for incorporation of normal dTTP and dGTP opposite templates A and C respectively (1.6 μM and 3.2 μM). The apparent *K*_m_ value for incorporation of 8-oxo-dGTP opposite template C was 212 μM.

**Table 1:** Kinetic parameters for 8-oxo-dGTP insertion catalysed by pol III^*^

Template base/dNTP	*K*_m_ (μM)	Relative *V*_max_	*V*_max_*/K*_m_	*f*_inc_
A/dTTP	1.6 ± 0.4	2.9 ± 0.23	1.8	1
A/8-oxo-dGTP	2.5 ± 0.82	0.26 ± 0.03	0.1	0.056
C/dGTP	3.2 ± 0.72	5.6 ± 0.47	1.75	1
C/8-oxo-dGTP	212 ± 45.7	1.1 ± 0.1	0.005	0.0029

### Excision of 8-oxo-dG at the end of the primer

**Fig. 3:**
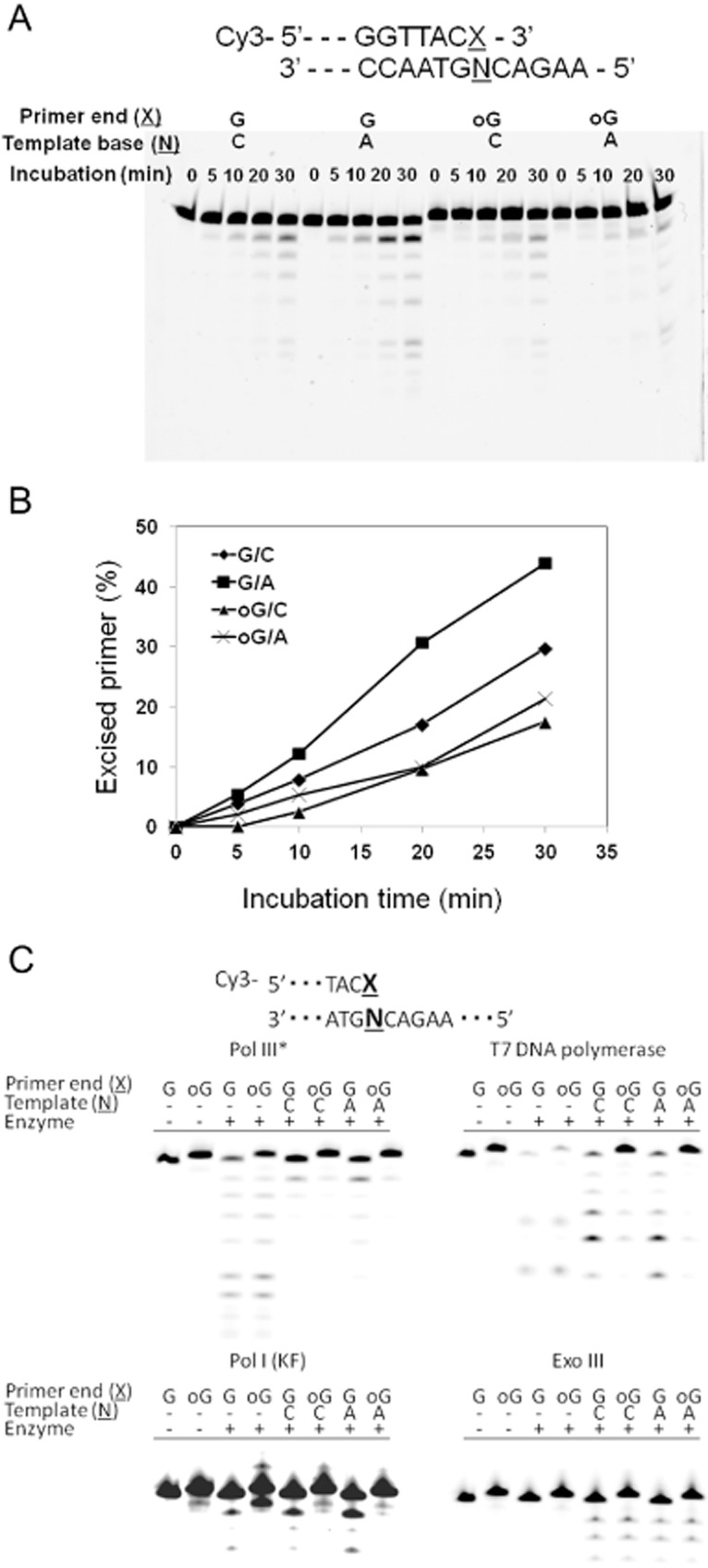
A. Exonuclease digestion of primers by pol III*. The Cy3-labelled 19-mer primer having G or 8-oxo-G at the 3′-termini/36-mer template having C or A at the position N (0.1 μM) were incubated with pol III* (1 nM) for 5, 10, 20 or 30 min at 25°C. The products were analysed by denaturing polyacrylamide gel electrophoresis and visualized by the Molecular Imager. oG represents 8-oxo-G. B. Time course of digestion of primers by pol III*. Four types of primer/template DNA having G/C, G/A, 8-oxo-G/C or 8-oxo-G/A at the termini were incubated with pol III* (1 nM) for 5, 10, 20 or 30 min at 25°C and the percentage of the digested primer DNA was plotted. C. Excision of 8-oxo-dGMP at the end of the primer by pol III*, T7 pol, pol I (KF) and exo III. The 19-mer primer/36-mer template DNA (0.1 μM) or the 19-mer primer DNA alone (0.1 μM) was incubated with pol III* (1 nM) at room temperature, T7 pol (0.0001 unit μl^−1^), pol I (KF)(0.001 unit μl^−1^) or exo III (0.0001 unit μl^−1^) at 37°C for 10 min without dNTP. The products were analysed as described in the legend to A.

The incorporated 8-oxo-dG opposite template A forms a mismatch, which is usually excised as an improper base by proofreading activities of pols. Thus, we examined whether incorporated 8-oxo-dGMP is excised by the proofreading activity of pol III* (Fig. 3A). Strikingly, 8-oxo-dGMP was not excised from the primer DNA regardless of the pairing template base of C or A efficiently. In contrast, pol III* effectively excised terminal normal dGMP incorrectly pairing with template A, and even correctly pairing with template C. When we plotted percentage of digestion of primer DNA periodically, it became evident that the order of primer most rapidly digested was primer having G/A mismatch at the termini > primer having G/C > primer having 8-oxo-G/A = primer having 8-oxo-G/C (Fig. 3B). Single-stranded DNA having 8-oxo-dGMP at the termini was rapidly degraded by pol III* as well as that having normal dGMP (Fig. 3C). For comparison, we conducted similar assays with other enzymes, i.e. T7 pol, *E. coli* pol I (KF) and exonuclease III. T7 pol displayed similar digesting patterns to those with pol III*. It could not effectively excise 8-oxo-dGMP at primer termini regardless of the template bases although it excised terminal normal dGMP incorrectly pairing with template A and less effectively correctly pairing normal dGMP with template C. It digested single-stranded DNA having 8-oxo-dGMP at the termini effectively. Pol I (KF) poorly excised 8-oxo-dGMP at primer termini as pol III* and T7 pol. In contrast to pol III* and T7 pol, pol I (KF) did not digest single-stranded DNA having 8-oxo-dGMP at the termini. Exonuclease III digested primer DNA having terminal 8-oxo-dGMP as well as normal dGMP. It did not digest single-stranded DNA regardless of the presence or the absence of 8-oxo-dGMP at the termini.

### Extension from dG or 8-oxo-dG at the end of a primer

**Fig. 4:**
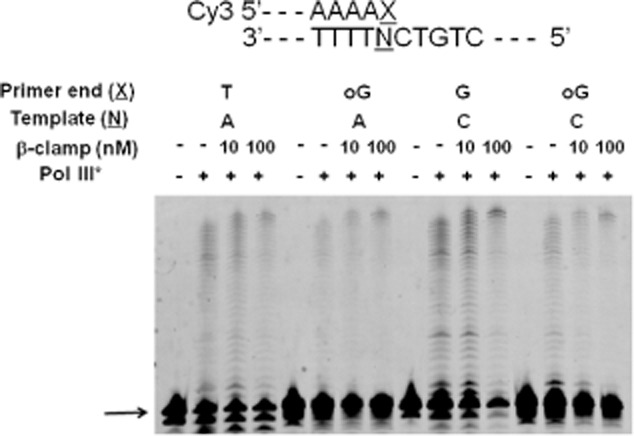
Standing start extension of primers having 8-oxo-G at the termini by pol III* with or without β clamp. Cy3-labelled 35-mer primer (X = T, G or 8-oxo-G) with 100-mer template DNA (N = A or C) having biotin/streptavidin at both ends (20 nM) was incubated with pol III* (10 nM) and β clamp (0, 10 or 100 nM) in the presence of 100 μM dNTPs. The reaction mixtures were incubated at room temperature for 1 min. The samples were analysed by denaturing polyacrylamide gel electrophoresis and visualized by the Molecular Imager. The alphabets shown in the figure represent: X, primer terminal base; N, template base; A, adenine; C, cytosine; G, guanine; T, thymine; oG, 8-oxo-G. The arrow indicates the position of primer. Although we have purified the primer DNA, there appears shorter primer DNAs, which were present below the position of the primer.

**Fig. 5:**
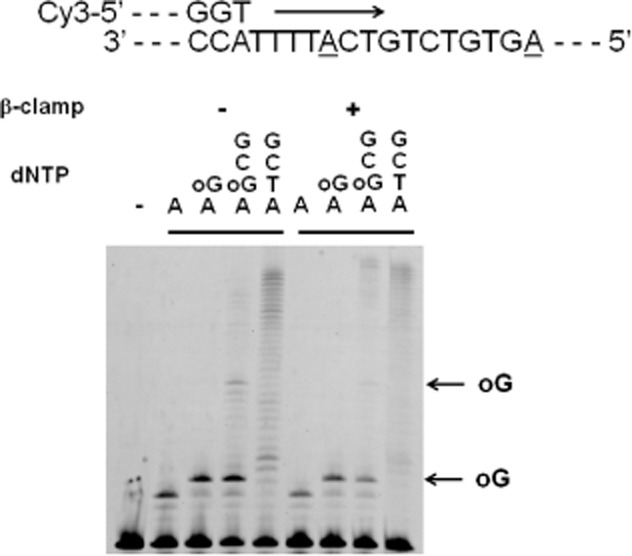
Incorporation of 8-oxo-dGTP and extension by pol III* with or without β clamp under running start conditions. The 30-mer primer/100-mer streptavidin bound template (sequences 2, 20 nM) were incubated with pol III* (10 nM) with or without β clamp (100 nM) in the presence of indicated dNTPs (100 μM each) for 1 min at 25°C. The samples were analysed by denaturing polyacrylamide gel electrophoresis and visualized by the Molecular Imager. The alphabets shown in the figure represent: A, dATP; C, dCTP; G, dGTP; T, dTTP; oG, 8-oxo-dGTP.

Primer DNA having incorporated 8-oxo-dGMP at the termini has to be extended. Otherwise, it will induce DNA strand breaks. Next, we examined whether primer DNA having 8-oxo-dGMP at the termini was extended by pol III* *in vitro*. We also examined the effects of addition of the β subunit to pol III* on the extension reactions. For this purpose, we prepared template DNA having biotin/streptavidin at both ends (Fig. S2). The terminal streptavidin prevents the β subunit from falling off from the template/primer DNA. In standing start experiments, pol III* extended primer DNA having 8-oxo-dG at the termini and the extension was substantially enhanced by the addition of the β subunit (Fig. 4). The primer/template DNA having 8-oxo-dGMP pairing with template A appeared to be slightly more effectively extended compared with the primer/template DNA having 8-oxo-dGMP pairing with template C. In running start experiments where 8-oxo-dGMP was incorporated into DNA during DNA synthesis, pol III* extended primer DNA upon incorporation of 8-oxo-dGMP opposite template A (Fig. 5). In these reactions, addition of the β subunit substantially enhanced the extension reactions. It should be noted, however, that the extension reactions upon incorporation of 8-oxo-dGMP opposite template A were less efficient compared with the reactions upon incorporation of dTMP opposite template A even in the presence of the β subunit. As controls, we also examined the extension activity of pol I (KF) and pol IV with primers having 8-oxo-dG at the termini (Fig. S3). Pol I (KF) displayed potent extension activity with primers having terminal 8-oxo-dGMP pairing with template A or C. The primer DNA having 8-oxo-dGMP pairing with template A was more effectively extended by pol I (KF) than primer DNA having normal G pairing with template A. Pol IV exhibited moderate extension activity with primer DNA having 8-oxo-dGMP pairing with template A or C.

## Discussion

dNTP pool and DNA are continuously exposed to a variety of exogenous and endogenous genotoxic agents, including ROS, and incorporation of oxidized dNTPs into DNA is a source of spontaneous mutagenesis and carcinogenesis (Ames and Gold, 1991). Here, we provided genetic and biochemical evidence that a replicative pol of *E. coli*, i.e. pol III HE, may be involved in oxidative mutagenesis through misincorporation of an oxidized nucleotide, i.e. 8-oxo-dGTP, during DNA synthesis in the *mutT* background. Although deletion of the genes encoding pol I, pol II, pol IV and pol V reduced the mutation frequency by more than 50%, the resulting strain YG6376 having pol III alone exhibited more than 50 times higher spontaneous mutation frequency in the *mutT* background than the wild-type strain (Fig. 1 and Table S1). Pol III* incorporated 8-oxo-dGTP opposite template A about 20 times more effectively than opposite template C *in vitro* (Fig. 2 and Table 1). Genetic analyses also suggest that 8-oxo-dGTP is preferentially incorporated opposite template A in the *mutT* background *in vivo* (Fowler *et al*., 2003). This biased specificity incorporating 8-oxo-dGTP opposite template A is reminiscent of that of X-family or Y-family pols involved in DNA repair and translesion DNA synthesis (Katafuchi and Nohmi, 2010). Mammalian pol β, a representative of X-family pols, incorporates 8-oxo-dGTP opposite template A and opposite template C at a ratio of 24:1 (Miller *et al*., 2000). Human pol η, a Y-family pol, preferentially incorporates 8-oxo-dGTP opposite template A at 60% efficiency of normal dTTP incorporation (Shimizu *et al*., 2007). Interestingly, α subunit, the catalytic subunit of pol III HE of *E. coli* and *Thermus aquaticus*, is structurally related to mammalian pol β, but not to mammalian replicative pols such as pol δ or pol ε, which are B-family enzymes (Bailey *et al*., 2006; Lamers *et al*., 2006). Therefore, we speculate that the structural similarity of the α subunit of pol III HE to mammalian pol β might be the structural basis for the preferential incorporation of 8-oxo-dGTP opposite A in template DNA.

In general, 8-oxo-dGTP is a poor substrate for most of pols (Nohmi *et al*., 2005; Katafuchi and Nohmi, 2010). For example, the efficiency of incorporation of 8-oxo-dGTP by pol δ involved in the chromosome replication in mammalian cells is more than 10^4^-fold lower than that of incorporation of normal dGTP, and the enzyme prefers to incorporate 8-oxo-dGTP opposite template C (Einolf and Guengerich, 2001). 8-oxo-dGTP is poorly incorporated into DNA by T7 pol exo^−^, HIV reverse transcriptase, *E. coli* pol II and pol I (KF) exo^−^ as well (Einolf *et al*., 1998). In contrast, pol III* incorporates 8-oxo-dGTP opposite template A at about 5% efficiency of normal dTTP incorporation (Table 1). We suggest that the erroneous and efficient incorporation of 8-oxo-dGTP opposite template A by pol III HE may account for the extremely high spontaneous mutations in the *mutT* mutants of *E. coli*.

At first, we expected that pol IV and pol V might be responsible for the misincorporation of 8-oxo-dGTP into DNA during DNA replication in the *mutT* background. This is because pol IV and pol V are involved in mutagenesis through incorporation of oxidized dNTPs into DNA in a Δ*sod*Δ*fur* background of *E. coli* (Yamada *et al*., 2006). In the mutants, intracellular ROS levels are extremely elevated, and SOS responses are fully induced (Nunoshiba *et al*., 1999; 2002). Therefore, expression levels of pol IV and pol V are highly elevated. In contrast, in the *mutT* mutants, no SOS responses are induced (Janion *et al*., 2003). Thus, it is expected that the expression of pol IV and pol V is constitutive levels. The different status of SOS induction might explain the different contribution of SOS-inducible Y-family pols to oxidative mutagenesis in Δ*sod*Δ*fur* and *mutT* strains although oxidized dNTPs are deeply involved in the mutagenic processes.

**Fig. 6:**
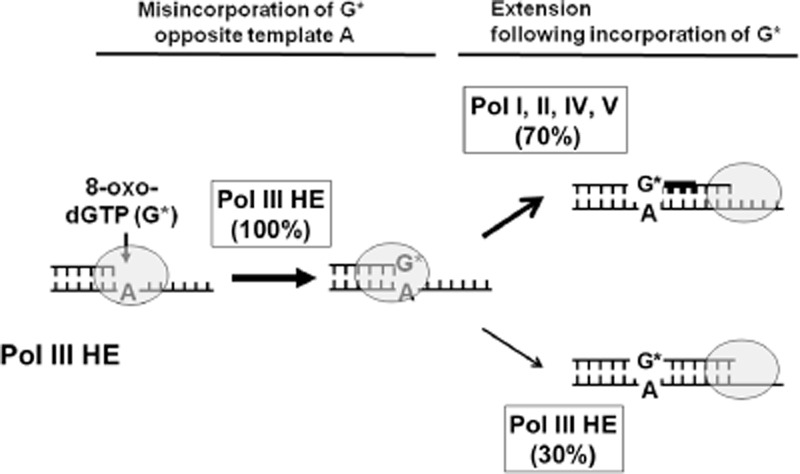
MutT mutator phenotype: a multi-polymerase affair. Pol III HE (oval) incorporates 8-oxo-dGTP (G*) opposite template A during the chromosomal replication. We suggest that while pol III accounts for nearly all 8-oxo-dGTP incorporation opposite template A, it only extends ≍ 30% of them, the remaining 70% being extended by the combined action of pol I, pol II, pol IV or pol V (based on data from Fig. 1). We also speculate that the roles of the auxiliary pols might be redundant because the mutation frequency was significantly reduced only when three or four auxiliary pols are deleted (Fig. 1). Following a short patch (a thick line) of DNA synthesis by the auxiliary pols, pol III HE will resume chromosomal replication.

It appears, however, that pols other than pol III play roles in the high spontaneous mutations in the *mutT* background. This is because the mutant lacking all four DNA polymerases (YG6376) exhibited a 70% reduction in mutability compared with *mutT* (Fig. 1 and Table S1). Single deletions of each of the genes encoding the auxiliary pols did not reduce the mutation frequency. Only in the cases where three or four pols are absent, there was a significant reduction of the *mutT* mutator phenotype. Thus, pol I, pol II, pol IV and pol V might have redundant roles in the mutagenesis. One possible explanation for the redundant role is that they might play roles in extension of primer DNA upon incorporation of 8-oxo-dGMP into DNA by pol III HE (Fig. 6). This speculation is based on the results that extension of primer upon incorporation of 8-oxo-dGMP opposite template A by pol III HE is less efficient compared with the incorporation of normal dTMP opposite template A (Fig. 5). In addition, pol I and pol IV possess ability to extend primer DNA having 8-oxo-dGMP opposite template A at the termini (Fig. S3). Hence, the auxiliary pols might play roles in the extension step, thereby enhancing the mutagenesis in the *mutT* background. Another, but not exclusive, alternative would be that the presence of pol I, pol II, pol IV and pol V might affect the efficiency of pol III HE incorporating 8-oxo-dGTP into DNA during the chromosome replication. It is well known that all five pols in *E. coli* interact with the β subunit and compete to take over the primer termini (Lopez de Saro *et al*., 2003; Burnouf *et al*., 2004). Thus, it seems possible that the absence of the auxiliary pols might affect the interactions of pol III with the β subunit, which in turn affects the efficiency of pol III HE incorporating 8-oxo-dGTP during DNA synthesis. It is reported that processivity factors such as eukaryotic PCNA and β subunit of *E. coli* affect the processivity and specificity of pols (Bloom *et al*., 1997; Maga *et al*., 2007).

Our biochemical results indicated that pol III*, T7 pol and pol I (KF) could not excise incorporated 8-oxo-dGMP from the primer effectively even when the oxidized dGMP was paired with template A (Fig. 3). Previous genetic analyses also suggest that the proofreading activity of pol III HE has little effect on the *mutT* mutator phenotype (Fowler *et al*., 1992). In pol III HE, the proofreading 3′ to 5′ exonuclease exists as a separate subunit ε, which binds to the catalytic subunit (McHenry, 2011). In contrast, exonucleases and polymerases exist in separate domains in single polypeptides of T7 pol and pol I (KF) (Beese *et al*., 1993; Doublie *et al*., 1998). Because pol III* and T7 pol can digest the single-stranded DNA having 8-oxo-dGMP and exonuclease III digested double-stranded DNA having 8-oxo-dGMP at the 3′-termini (Fig. 3), the exonucleases in the pols and exonucleases III appear to have the ability to hydrolyse phosphodiester bonds between the terminal 8-oxo-dGMP and the second terminal dNMP in the primer strands. Therefore, we speculate that the reason for the poor excision of 8-oxo-dGMP from the 3′-termini of primer strands by pol III* and T7 pol might be inefficient transfer of the primer strands having 8-oxo-dGMP from the polymerase domain (or subunit) to the exonuclease domain (or subunit) even when 8-oxo-dGMP pairs with template A. In contrast, pol I (KF) did not digest single-stranded DNA having 8-oxo-dGMP at the termini. Therefore, in the case of pol I (KF), the poor proofreading against 8-oxo-dGMP paired with template A or C may be due to its weak or defective nuclease activity against single-stranded DNA having 8-oxo-dGMP at the terminus. It might be interesting to investigate the structural and biochemical reasons for the poor excision of 8-oxo-dGMP at the 3′-termini of primers by the pols.

It may be counterintuitive that pol III HE, the replicative pol, which is supposed to be very accurate, positively produces errors during the chromosome replication if *mutT* is inactivated. One plausible explanation is that pol III HE incorporates the oxidized dGTP into DNA, thereby enhancing mutagenesis to generate progenies that can adapt to the stressful environments, when the bacteria are exposed to oxidative stress and the *mutT* gene is inactivated. The original habitat of *E. coli* is intestine, which is strictly anaerobic. Therefore, aerobic culture conditions may be somewhat stressful to *E. coli*. A precedent for such enhanced mutagenesis in stressful conditions is the SOS-induced mutagenesis where *E. coli* induces error-prone pol IV and pol V and enhances mutagenesis when DNA is damaged by ultraviolet light or other DNA-damaging agents (Echols, 1981; Friedberg *et al*., 2002). High mutation rates may be detrimental to individual bacterium but may be beneficial for the whole population because the high mutation rates may lead to generation of mutant progenies that can survive under the stressful conditions. Other C-family pols from organisms originally living in anaerobic conditions might have evolved in a manner to incorporate 8-oxo-dGTP into DNA effectively as in the case of *E. coli* pol III HE.

In summary, we presented genetic and biochemical evidence that suggests that the replicative pol of *E. coli*, i.e. pol III HE, effectively and incorrectly incorporates oxidized dGTP opposite template A during the chromosome replication *in vivo*. The auxiliary pols appear to help the erroneous DNA replication by pol III HE in the *mutT* background. The specificity and efficiency of incorporation of 8-oxo-dGTP into DNA by pol III HE are marked contrast with those of replicative pols in mammals, e.g. pol δ, which incorporate 8-oxo-dGTP into DNA very poorly. The erroneous nature of pol III HE, which is uniquely present in eubacteria, might explain the extremely high spontaneous mutations of the *mutT* background in *E. coli* compared with the moderate mutator effects of mammalian counterparts lacking enzymes with 8-oxo-dGTP hydrolysing activities such as MTH1.

## Experimental procedures

### Materials

Pol III* was purified as described (Fujii and Fuchs, 2004). Deoxyribonucleoside triphosphates (ultrapure grade) and 8-oxo-dGTP were purchased from Amersham Pharmacia Biotech (Buckinghamshire, UK) and TriLink BioTechnologies (San Diego, CA, USA) respectively. Oligonucleotides were synthesized and purified twice by high-performance liquid chromatography (BEX, Tokyo, Japan). Primers were annealed to templates at a 1:1 molar ratio. For primer extension and kinetic studies of nucleotide incorporation, two types of DNA substrates were used. The set of the 18-mer primer/36-mer template named sequences 1 was as follows: 5′-CGCGCGAAGACCGGTTAC-3′ (18-mer primer) and 3′-GCGCGCTTCTGGCCAATGNCAGAATTCCTAGGGAAG-5′ (36-mer template, where N = A, C, G or T). The set of the 30-mer primer/100-mer template named as sequences 2 used for measuring the incorporation frequencies was as follows: 5′-GTACCGCCACCCTTAGAACCGGTGTTTGGT-3′ (primer; 30-mer) and 3′-GGCCTTATCCACATAGTGGCATGAGTCCTCCAAATCATGGCGGTGGGAATCTTGGCCACAAACCATTTTXCTGTCTGTGACTCGTTCAGGCTATTACTGA-5′ [template; 100-mer, X (= A or C) is the target site]. The same 100-mer oligonucleotide with biotin was synthesized and purified twice by high-performance liquid chromatography (Tsukuba Oligo Service, Tsukuba, Japan).

### Strain construction

All the strains and plasmids used in this study are listed in Table 2. P1 transduction was conducted at 37°C. When strains lacking pol I Klenow fragment (ΔKF) were used as either recipients or donors, the transduction was conducted at 30°C.

**Table 2:** Strains and plasmids used in this study

Strains	Genetic characteristics	Sources
AB1157	F^−^ *thr1 ara14 leuB6 proA2 lacG1 tsx33 supE44 galK2 hisG4 rfbD1 mgl51 rpsL31 xyl5 mtl1 argE3 thi1* λ^−^ *rac*^−^	Laboratory stock
V355	F^−^ *lac-3350 galK2*(*O*^c^) *galT22* λ*^−^ recD1014*(Nuc^−^) *IN(rrnD-rrnE)1 rpsL179*(str^R^)	Shevell *et al*. (1988)
JW0059	F^−^ Δ*polB770*::*kan* Δ(*araD–araB*)*567* Δ*lacZ4787*(::*rrnB-3*) λ^−^ *rph-1* Δ(*rhaD–rhaB*)*568 hsdR514*; Km^R^	Keio Collection, NBRP
YG2004	The same as V355, but deficient in *mutT* with Km^R^ cassette insertion; Km^R^, Δ*mutT*	This study
YG6156	The same as AB1157, but deficient in *mutT* with Km^R^ cassette insertion; Km^R^, Δ*mutT*	This study
HRS7052	Deficient in a part of *polA* gene, thus lacking both 3′–5′ exonuclease and polymerase activities; Cm^R^, ΔKF	Wagner and Nohmi (2000)
YG6162	The same as AB1157, but deficient in *dinB* with in frame deletion; Δ*dinB*	Salem *et al*. (2009)
YG6167	The same as YG6162, but deficient in *mutT* with Km^R^ cassette insertion; Km^R^, Δ*dinB* Δ*mutT*	This study
YG6168	The same as AB1157, but deficient in *umuDC* with *ermGT* insertion, Δ*umuDC*	Salem *et al*. (2009)
YG6174	The same as YG6168, but deficient in *mutT* with Km^R^ cassette insertion; Km^R^, Δ*umuDC* Δ*mutT*	This study
YG6171	The same as YG6168, but deficient in *dinB* with in frame deletion, Δ*umuDC* Δ*dinB*	Salem *et al*. (2009)
YG6172	The same as YG6171, but deficient in *mutT* with Km^R^ cassette insertion; Km^R^, Δ*umuDC* Δ*dinB* Δ*mutT*	This study
YG6343	The same as AB1157, but deficient in a part of *polA* gene, thus lacking both 3′–5′ exonuclease and polymerase activities *with* Cm^R^ cassette insertion; Cm^R^, ΔKF	This study
YG6347	The same as YG6343, but deficient in *mutT* with Km^R^ cassette insertion; Cm^R^; Km^R^, ΔKF Δ*mutT*	This study
YG6371	The same as YG6162, but deficient in a part of *polA* gene, thus lacking both 3′–5′ exonuclease and polymerase activities *with* Cm^R^ cassette insertion; Cm^R^, Δ*dinB* ΔKF	This study
YG6372	The same as YG6371, but deficient in *mutT* with Km^R^ cassette insertion; Cm^R^; Km^R^, Δ*dinB* ΔKF Δ*mutT*	This study
YG6375	The same as YG6171, but deficient in a part of *polA* gene with Cm^R^ cassette insertion as HRS7052 and deficient in *polB* gene with in frame deletion, Cm^R^; Δ*umuDC* Δ*dinB* ΔKF Δ*polB*	This study
YG6376	The same as YG6375, but deficient in *mutT* with Km^R^ cassette insertion; Cm^R^; Km^R^, Δ*umuDC* Δ*dinB* Δ*polB* ΔKF Δ*mutT*	This study
YG6377	The same as AB1157, but deficient in *polB* with in frame deletion; Δ*polB*	This study
YG6378	The same as YG6377, but deficient in *mutT* with Km^R^ cassette insertion; Km^R^, Δ*polB* Δ*mutT*	This study
YG6381	The same as YG6168, but deficient in *polB* with in frame deletion; Δ*umuDC* Δ*polB*	This study
YG6382	The same as YG6171, but deficient in *polB* with in frame deletion; Δ*umuDC* Δ*dinB* Δ*polB*	This study
YG6385	The same as YG6382, but deficient in *mutT* with Km^R^ cassette insertion; Km^R^, Δ*umuDC* Δ*dinB* Δ*polB* Δ*mutT*	This study
YG6383	The same as YG6171, but deficient in a part of *polA* gene with Cm^R^ cassette insertion as HRS7052; Cm^R^, Δ*umuDC* Δ*dinB* ΔKF	This study
YG6386	The same as YG6383, but deficient in *mutT* with Km^R^ cassette insertion; Cm^R^, Km^R^, Δ*umuDC* Δ*dinB* ΔKF Δ*mutT*	This study
YG6384	The same as YG6381, but deficient in a part of *polA* gene with Cm^R^ cassette insertion as HRS7052, Cm^R^; Δ*umuDC* Δ*polB* ΔKF	This study
YG6387	The same as YG6384, but deficient in *mutT* with Km^R^ cassette insertion; Cm^R^; Km^R^, Δ*umuDC* Δ*polB* ΔKF Δ*mutT*	This study
YG6379	The same as YG6371, but deficient in *polB* with in frame deletion; Cm^R^, Δ*dinB* ΔKF Δ*polB*	This study
YG6380	The same as YG6379, but deficient in *mutT* with Km^R^ cassette insertion; Cm^R^, Km^R^, Δ*dinB* ΔKF Δ*polB* Δ*mutT*	This study

Plasmids		
pYG703	Plasmid pUC19, but has the *mutT* gene	This study
pYG704	The same as pYG703, but has *mutT::*Km^R^	This study
pCP20	pCP20 has the yeast Flp recombinase gene, *FLP*, chloramphenicol and ampicillin resistance genes, and temperature-sensitive replication; Ap^R^, Cm^R^	CGSC

To construct *polA*^−^ derivative, the Δ*klenow*::chloramphenicol resistance (Cm^R^) gene (*cat*) allele of strain HRS7052 (Wagner and Nohmi, 2000), which was constructed by Dr H. Iwasaki (Tokyo Institute of Technology, Japan), was transferred to AB1157 by P1 transduction. The resultant strain was named as YG6343, which lacked pol and 3′ to 5′ exonuclease of pol I but retained 5′ to 3′ exonuclease activity. YG6371 (ΔKFΔ*dinB*) was constructed by P1 transduction using HRS7052 as a donor and YG6162 (Δ*dinB*, see below) as a recipient. Similarly, YG6383 (ΔKFΔ*dinB*Δ*umuDC*), YG6384 (ΔKFΔ*polB*Δ*umuDC*) and YG6375 (ΔKFΔ*polB*Δ*dinB*Δ*umuDC*) were constructed by P1 transduction with YG6171 (Δ*dinB*Δ*umuDC*), YG6381 (Δ*polB*Δ*umuDC*, see below) and YG6382 (Δ*polB*Δ*dinB*Δ*umuDC*, see below) respectively.

To construct *polB*^−^ derivative, *polB*::kanamycin resistance (Km^R^) gene (*kan*) was transferred from JW0059 into a recipient AB1157, YG6171 (Δ*dinB*Δ*umuDC*; Salem *et al*., 2009), YG6371 (ΔKFΔ*dinB*) or YG6168 (Δ*umuDC*; Salem *et al*., 2009) by P1 transduction, and the Km^R^ colonies were selected. The Km^R^ strains were transformed with pCP20 [ampicillin resistance (Ap^R^), Cm^R^], which encodes a yeast FLP recombinase. The Cm^R^ transformants were selected at 30°C since the replication origin of the plasmid was temperature-sensitive. In the case of YG6371 (ΔKFΔ*dinB*), Ap^R^ transformants were selected. A few colonies were picked up and streaked on a fresh plate, and then incubated at 43°C. An expression of the FLP was induced, which resulted in removal of the Km^R^ cassette whose both ends had recognition sites for the FLP recombinase. The high temperature also makes pCP20 cured from the cells. The obtained colonies were confirmed for their sensitivity to Cm (except for ΔKF strain), Ap and Km and also the size of the bands by PCR analysis (Table S2). The resultant strains were designated as YG6377 (Δ*polB*), YG6382 (Δ*polB*Δ*dinB*Δ*umuDC*), YG6379 (ΔKFΔ*polB*Δ*dinB*) and YG6381 (Δ*polB*Δ*umuDC*) respectively. They had no drug resistance maker for the *polB* deletion.

To construct *mutT*-deficient strains, we cloned the *mutT* gene from Kohara library (Kohara *et al*., 1987) into pUC19, then designated as pYG703. Inserting 1.3 kb EcoRI cassette carrying the Km^R^ gene into EcoRI site of pYG703, the clone in which transcription direction of *mutT* is opposite to the *kan* gene was selected and named as pYG704. After digestion of pYG704 with KpnI, the linear fragment was isolated and introduced into strain V355, which lacks *recD*, to obtain the clone whose *mutT* was replaced with Δ*mutT*::*kan*. The resultant strain was designated as YG2004, which was used to transfer Δ*mutT*::*kan* to strain AB1157 and the pol-defective derivatives by P1 transduction. The *mutT*::*kan* derivative of AB1157 was designated as YG6156 (AB1157Δ*mutT*). Other host strains for the P1 transduction were YG6162 (Δ*dinB*; Salem *et al*., 2009), YG6168 (Δ*umuDC*), YG6171 (Δ*dinB*Δ*umuDC*), YG6377 (Δ*polB*) and YG6382 (Δ*polB*Δ*dinB*Δ*umuDC*). The resultant strains were designated as YG6167 (Δ*dinB*Δ*mutT*), YG6174 (Δ*umuDC*Δ*mutT*), YG6172 (Δ*dinB*Δ*umuDC*Δ*mutT*), YG6378 (Δ*polB*Δ*mutT*), YG6385 (Δ*polB*Δ*dinB*Δ*umuDC*Δ*mutT*) respectively. In addition, the Δ*mutT*::*kan* allele was transferred from YG2004 to strains YG6343 (ΔKF), YG6371 (ΔKFΔ*dinB*), YG6383 (ΔKFΔ*dinB*Δ*umuDC*), YG6384 (ΔKFΔ*polB*Δ*umuDC*), YG6375 (ΔKFΔ*polB*Δ*dinB*Δ*umuDC*) and YG6379 (ΔKFΔ*polB*Δ*dinB*) at 30°C, and the resultant strains were designated as YG6347 (ΔKFΔ*mutT*), YG6372 (ΔKFΔ*dinB*Δ*mutT*), YG6386 (ΔKFΔ*dinB*Δ*umuDC*Δ*mutT*), YG6387 (ΔKFΔ*polB*Δ*umuDC*Δ*mutT*), YG6376 (ΔKFΔ*polB*Δ*dinB*Δ*umuDC*Δ*mutT*) and YG6380 (ΔKFΔ*polB*Δ*dinB*Δ*mutT*) respectively.

To confirm proper replacements in the chromosome, each constructed strain was subject to polymerase chain reactions with primers designed to display different amplification sizes when gene replacements successfully occur (Table S2).

### Mutation assay

To determine mutation frequency, acquisition of resistance to rifampicin was used in fluctuation analysis. A fresh single colony was picked from LB agar and grown overnight in a tube at 37°C with aeration in LB medium and 10 tubes were prepared for each strain. The culture was diluted 10^6^ times in fresh LB medium to achieve no mutants in one culture. The cultures were grown at 37°C with aeration (on a roller drum) to saturation (16 h), and 50 μl of aliquot for each tube was plated on a LB agar plate supplemented with 100 μg ml^−1^ rifampicin (Sigma, OH, USA). For viable cell count, three randomly selected cultures were serially diluted in cold saline and plated in LB agar without antibiotics. All plates were incubated at 37°C, except for the Klenow-deficient strains for which 30°C was used, for 24 h before counting colonies. We repeated the experiments three times for the determination of mutation frequency, which was calculated with median for 10 cultures divided by a mean value of viable cell count (Hasegawa *et al*., 2008).

### Primer extension assay

The enzyme reaction buffer containing 20 mM Tris-HCl (pH 7.5), 4% glycerol, 8 mM DTT, 80 µg ml^−1^ BSA, 2.5 mM ATP, 8 mM MgCl_2_, 100 μM 8-oxo-dGTP, 1 nM pol III* was incubated with 0.1 μM 5′-Cy3-primer/template (sequences 1) at room temperature (20 to 25°C) for 1 min. Reactions were terminated by adding 10 μl of stop solution (98% formamide, 10 mM EDTA, 10 mg ml^−1^ Blue Dextran). Samples were denatured at 100°C for 10 min, then applied to 15% denaturing polyacrylamide gel for electrophoresis and the patterns were visualized by the Molecular Imager FX Pro System (Bio-Rad, CA, USA).

### Kinetics analysis

For incorporation kinetics, 1 nM pol III*, 0.1 μM substrate (sequences 2) and 0.05–500 μM dNTPs were incubated at room temperature for 1 to 3 min in the reaction buffer written above. The reaction samples were subjected to 15% denaturing polyacrylamide gel. The gel band intensities were measured using the Molecular Imager FX Pro System and Quantity One software (Bio-Rad). The nucleotide incorporation efficiency opposite the target site was obtained by measuring *I_T_*^Σ^*/I*_*T*−1_, where *I_T_*^Σ^ represents the integrated gel band intensities of primers extended to the target site and beyond, and *I*_*T*-1_ is the integrated gel band intensity of primers extended to the site just prior to the target site (Creighton and Goodman, 1995; Bloom *et al*., 1997). For each DNA substrate, the rate of incorporation was plotted as a function of dNTP concentration, and the relative *V*_max_ and apparent *K*_m_ values were determined by nonlinear regression fitting using the SigmaPlot software (SigmaPlot Software Sciences, IL, USA). The relative *V*_max_ value is equal to the maximum value of *I_T_*^Σ^*/I*_*T*−1_. The frequency of incorporation (*f*_inc_) was calculated using the equation *f*_inc_ = (*V*_max_/*K*_m_)_incorrect_ / (*V*_max_/*K*_m_)_correct_. All values are means (± standard error) of three experiments.

### Assay for excision of 8-oxo-G-ended primer

The set of the 19-mer primer/36-mer template was basically the same as sequences 1 (see above). It was as follows: 5′-CGCGCGAAGACCGGTTACX-3′ (19-mer primer, where X = G or 8-oxo-G) and 3′-GCGCGCTTCTGGCCAATGNCAGAATTCCTAGGGAAG-5′ (36-mer template, where N = A or C). The primer whose 3′-terminus is 8-oxo-G was purchased from Tsukuba Oligo Service (Tsukuba, Japan). For the assay for excision of 8-oxo-G-ended primer, these primer/templates (0.1 μM) were incubated with 1 nM pol III* at 25°C for 5, 10, 20 or 30 min in the reaction buffer described above. The products were analysed by denaturing polyacrylamide gel electrophoresis and visualized by the Molecular Imager. Percentage of the amount of digested primers compared with that of the original primers was calculated. For comparison, the primer/template DNA or the primer DNA alone was incubated pol III* (1 nM) at room temperature, T7 pol (0.0001 unit μl^−1^), pol I (KF) (0.001 unit μl^−1^) or exo III (0.0001 unit μl^−1^, New England BioLabs) at 37°C for 10 min in a reaction mixture for each enzyme without dNTP. The products were analysed as described above.

### Assay for primer extension with β clamp

Primer extension with the β subunit was examined under two different conditions, i.e. standing start and running start experiments. In the standing start experiments, the primer was the same as that of sequences 2, but had extra five bases at the 3′-end, i.e. 5′-GTACCGCCACCCTTAGAACCGGTGTTTGGTAAAAX-3′ (35-mer, where X = T, G or 8-oxo-G). The template was the same as that of sequences 2 (100-mer), but had biotin/streptavidin at both ends (see Fig. S2). The primer/template DNA (20 nM), pol III* (10 nM) and four normal dNTPs (100 μM) were incubated for 1 min at 25°C. When the β subunit (10 or 100 nM) was included, the reaction mixture without dNTPs was pre-incubated for 10 min at 25°C and the reaction was started by addition of dNTPs. The reaction buffer and the methods to analyse the reaction products were the same as those described in the primer extension assay. In running start experiments, the primer (30-mer)/template (100-mer) DNA was the same as those of sequences 2, but the template DNA had A at the position of N and biotin/streptavidin at both ends. The primer/template DNA (20 nM), pol III* (10 nM) and dNTP(s) (100 μM) were incubated in the presence or the absence of the β subunit (100 nM) for 1 min at 25°C. In these experiments, four types of dNTP solution were used. Each contained dATP alone, dATP and 8-oxo-dGTP, dATP, 8-oxo-dGTP, dCTP and dGTP, or four normal dNTPs.
