# Supplementary material for: Escherichia coli DNA polymerase III is responsible for the high level of spontaneous mutations in mutT strains
Source: Mol Microbiol. 2012 Nov 1;86(6):1364–75. doi: 10.1111/mmi.12061 (PMC3556519; doi:10.1111/mmi.12061)
Supplement: Supplementary file 1 [file mmi0086-1364-SD1.pdf]

Supplement Table 1 Median mutation frequencies of *mutT* derivatives of *E. coli*

date	YG6376 ΔKFΔ*polB* Δ*dinB* Δ*umuDC* Δ*m*	YG6156(30℃)	date	YG6385 Δ*polB* Δ*dinB* Δ*umuDC* Δ*mutT*	YG6156(37℃)	date	YG6386 ΔKFΔ*dinB* Δ*umuDC* Δ*mutT*	YG6156(30℃)
120222	54	221	120502	211,155,169	196	120509	73,175,143	216
120328	79,112	228,287						
120611	53,69,79	206						
	* 74±20 (29±8%)	251±31		178±24 (91±12%)	196		130±43 (60±20%)	216

date	YG6387 ΔKFΔ*polB* Δ*umuDC* Δ*mut*	YG6156(30℃)	date	YG6380 ΔKFΔ*polB* Δ*dinB* Δ*mutT*	YG6156(30℃)	date	YG6372 ΔKFΔ*dinB* Δ*umuDC* Δ*mutT*	YG6156(30℃)
120510	132,124,119	216	110414	102	104	110414	157	104
			110421	180	154	110421	121	154
			110512	137	200	110512	118	200
	125±6 (58±3%)	216		140±32 (92±21%)	153±39		132±18 (86±12%)	153±39

date	YG6172 Δ*dinB* Δ*umuDC* Δ*mutT*	YG6156(37℃)	date	YG6347 Δ*KF* Δ*mutT*	YG6156(30℃)	date	YG6378 Δ*polB* Δ*mutT*	YG6156(37℃)
120626	280,249	256	101216	139	84	120322	176	189
120706	261	194	101219	146	103	120405	248,220	227
			110120	210	184	120612	251,271,278	265
	263±13 (117±6%)	225		165±32 (133±26%)	124±31		241±34 (106±15%)	227±31

date	YG6167 Δ*dinB* Δ*mutT*	YG6156(37℃)	date	YG6174 Δ*umuDC* Δ*mutT*	YG6156(37℃)
101028	137	116	101028	124	116
101111	107	114	101111	157	114
101118	142	105	101118	110	105
	129±16 (115±14%)	112±5		130±20 (116±18%)	112±5

date	YG6156 (30℃) Δ*mutT*
101216	84
101219	103
110120	184
110414	104
110421	154
110512	200
120222	221
120328	228,287
120509	216
120510	216
120611	206
	184±58

date	YG6156 (37℃) Δ*mutT*
101028	116
101111	114
101118	105
120322	189
120405	227
120502	196
120612	265
120626	256
120706	194
	185±57

date	AB1157(30℃)
110414	1
110421	1.2
110512	1.8
	1.3±0.3

All numbers are median frequency x 10^8

Relative values (percentage) of median frequency compared to the concurrent control values of YG6156 are presented within parentheses.

* Statistical difference by student t-test ($p<0.05$)

Supplement Table 2 Primers for PCR and the band sizes of the wild-type and mutant strains

gene	primer sequence	band size for wild type strains	band size for deficient strains
kle-F	5'-GAA GAT CGA TCC GAA AGT GCT G-3'	1.1 kb	no bands
kle-R	5'-TCC ACC AGC AAC GGC ACA TC-3'		
cat-F	5'-ATT CTT GCC CGC CTG ATG AAT G-3'	no bands	0.4 kb
cat-R	5'-CCA CTC ATC GCA GTA CTG TTG-3'		
polA-F	5'-TGACTAACAGCGCAGGCGAGC-3'	no bands	2.5 kb
cat-R	5'-CCA CTC ATC GCA GTA CTG TTG-3'		
polB-F	5'-AAA TGA CCG ACG CAG AAA TCA ACG-3'	2.8 kb	0.5 kb
polB-R	5'-AAA GGG CGC TAT GGT ACT GGA TGG-3'		
dinB-F	5'-GTC AGC ATG GGG ATA AAG TGG TGC AG-3'	1.2 kb	0.3 kb
dinB-R	5'-CAG CGA GAA TTC GAT GCA TAC AGT GAT AC-3'		
umuDC-F	5'-AAG AGA ACG AAA AAT CAG CAG C-3'	1.9 kb	2.3 kb
umuDC-R	5'-TCG GTA TTT TTA TCG CTT ACC TGA-3'		
mutT-F	5'-GTA TGG AAG CCG AGC GTT TAG CGC AAT TGC-3'	0.8 kb	2.2 kb
mutT-R	5'-TGA TCG ACC TCG GAG AAT GGG CTG CTG AAG-3'		

Legends for supplement Figures

Supplement Fig. 1 Efficiency of 8-oxo-dGTP incorporation by pol III*. Reaction mixture contained 30mer primer/100mer template (Sequences 2, 0.1 μM), pol III* (1 nM), dATP (50 μM) and various concentrations of dGTP, dTTP or 8-oxo-dGTP. Exact concentrations of dNTPs in Fig. 3 A and B were as follows. In Fig. 3A, dTTP and 8-oxo-dGTP concentrations were 0.05, 0.25, 0.5, 2.5, 5 or 10 μM. In Fig. 3 B, dGTP concentration was 0.05, 0.25, 0.5, 2.5, 5 or 10 μM and 8-oxo-dGTP concentration was 10, 50, 100, 200, 300 or 500 μM. The reaction mixtures were incubated for 1 min at room temperature, except that the mixtures were incubated for 3 min for 8-oxo-dGTP incorporation opposite template C (B). A control reaction for the running-start was incubated for 1 min with dATP (50 μM) alone. The position of the band for Cy3-labeled 30-mer primer is shown at the left side on the gel. The template sequences corresponding to the position of the bands for the extended primers are indicated on the right side of the gel.

Supplement Fig. 2 Binding of streptavidin to the oligonucleotide DNA. The Cy3-labeled 30mer primer/100mer template (Sequences 2, 1 μM) with or without biotin at the both ends were incubated with streptavidin (10 μM, Thermo

Scientific) at room temperature for 10 min. The samples were analyzed by native polyacrylamide-gel electrophoresis and visualized by the Molecular Imager as described in Experimental Procedures.

Supplement Fig. 3. Comparison of the extension from dG or 8-oxo-dG (oG) at the end of a primer using pol I (KF) (A) and pol IV (B). The set of the 19-mer primer/36-mer template (0.1 μM) was incubated with 0.0005 units/μL pol I (KF) or 10 nM pol IV for 10 min at 37°C in a reaction buffer for each enzyme with 100 μM each of dNTPs. The extended primers were separated by 15% denaturing polyacrylamide gel electrophoresis and visualized by Molecular Imager FX Pro system.

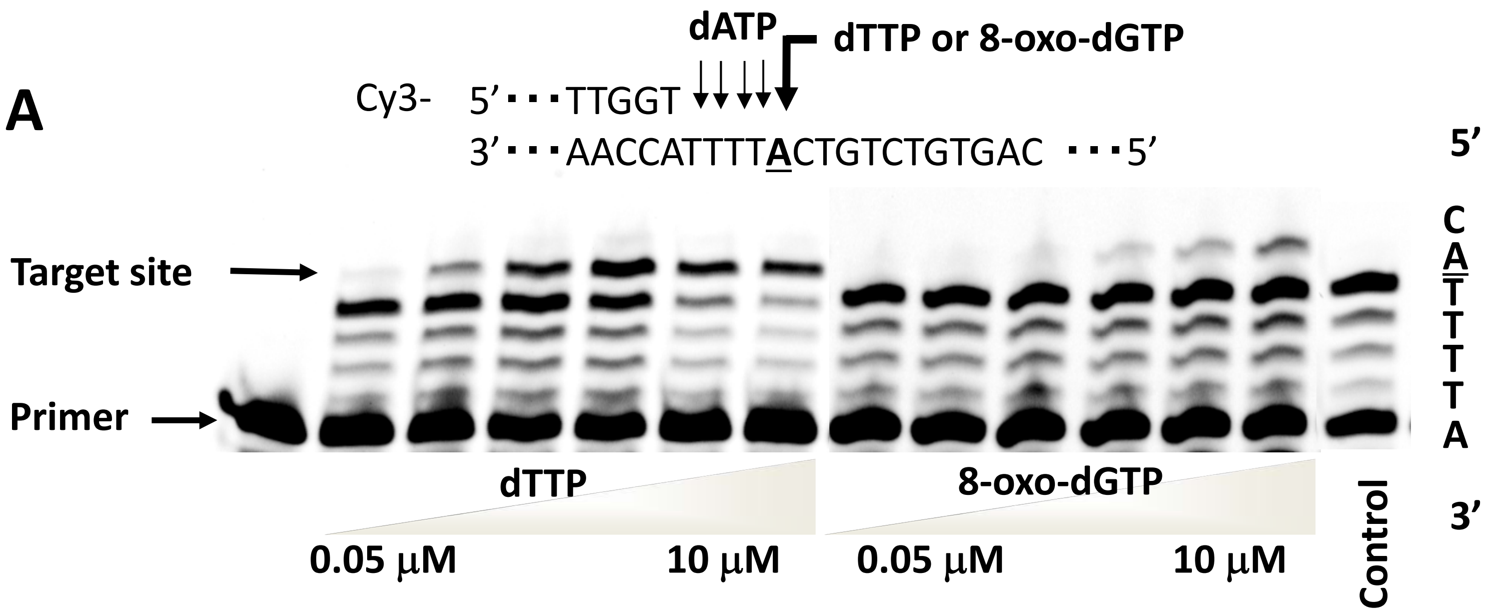

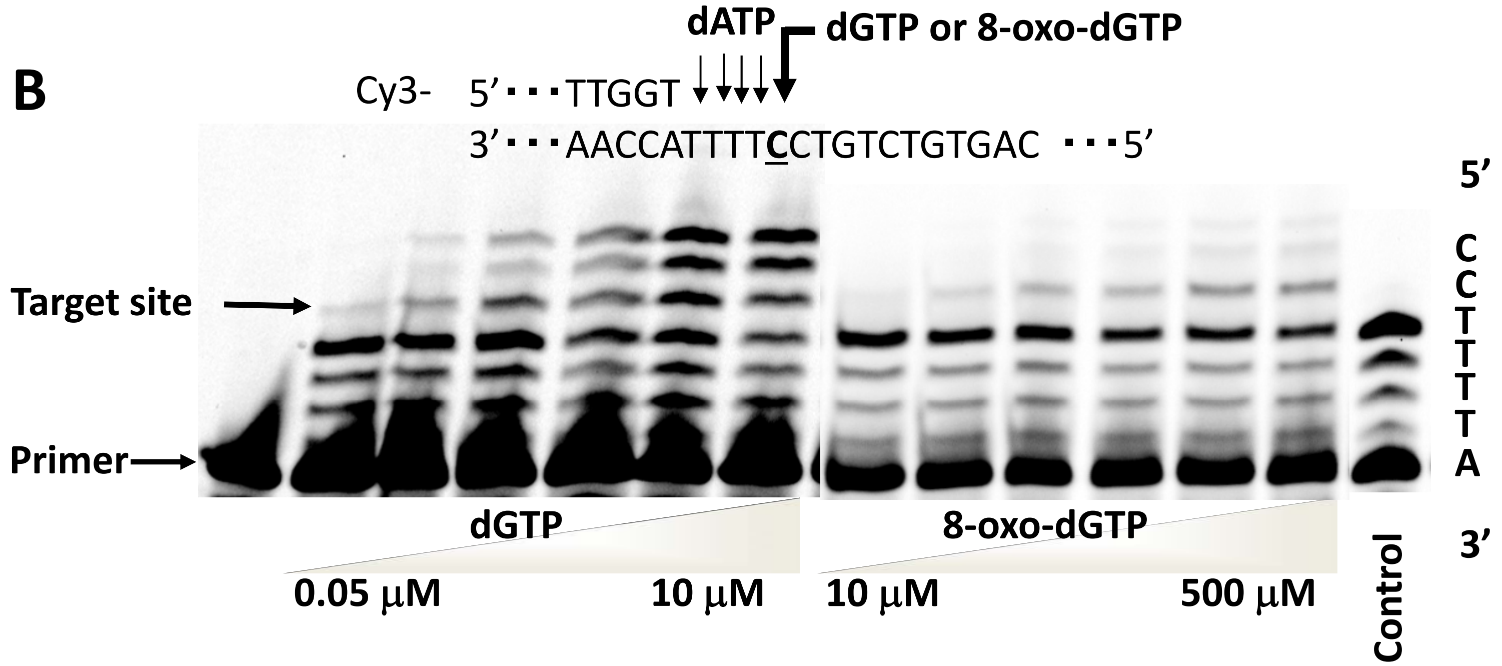

Supplement Fig. 1

Cy3-5’ - - - GGT
3’ - - - CCATTTTNCTGTCTGTGA - - - 5’

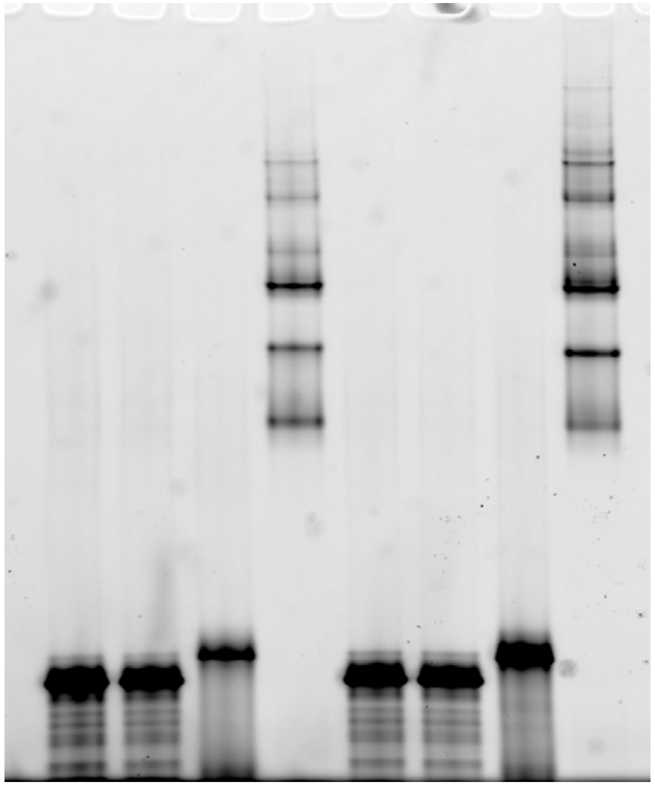

**biotinylation**	**-**	**-**	**+**	**+**	**-**	**-**	**+**	**+**
**streptavidin**	**-**	**+**	**-**	**+**	**-**	**+**	**-**	**+**
**Template (N)**			**A**				**C**	

Supplement Fig. 2

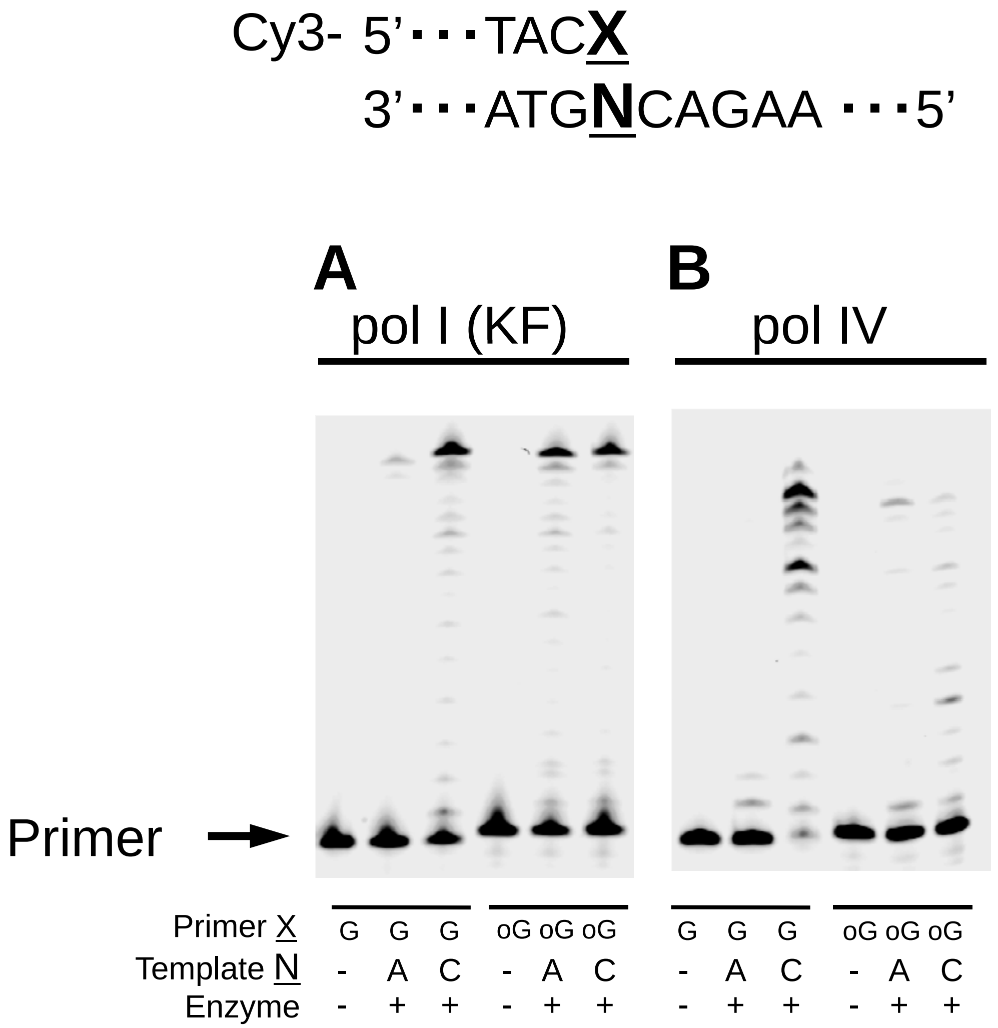

Supplement Fig. 3
