# Supplementary figures and images for: Escherichia coli DNA polymerase III is responsible for the high level of spontaneous mutations in mutT strains
Source: Mol Microbiol. 2012 Nov 1;86(6):1364–75. doi: 10.1111/mmi.12061 (PMC3556519; doi:10.1111/mmi.12061)

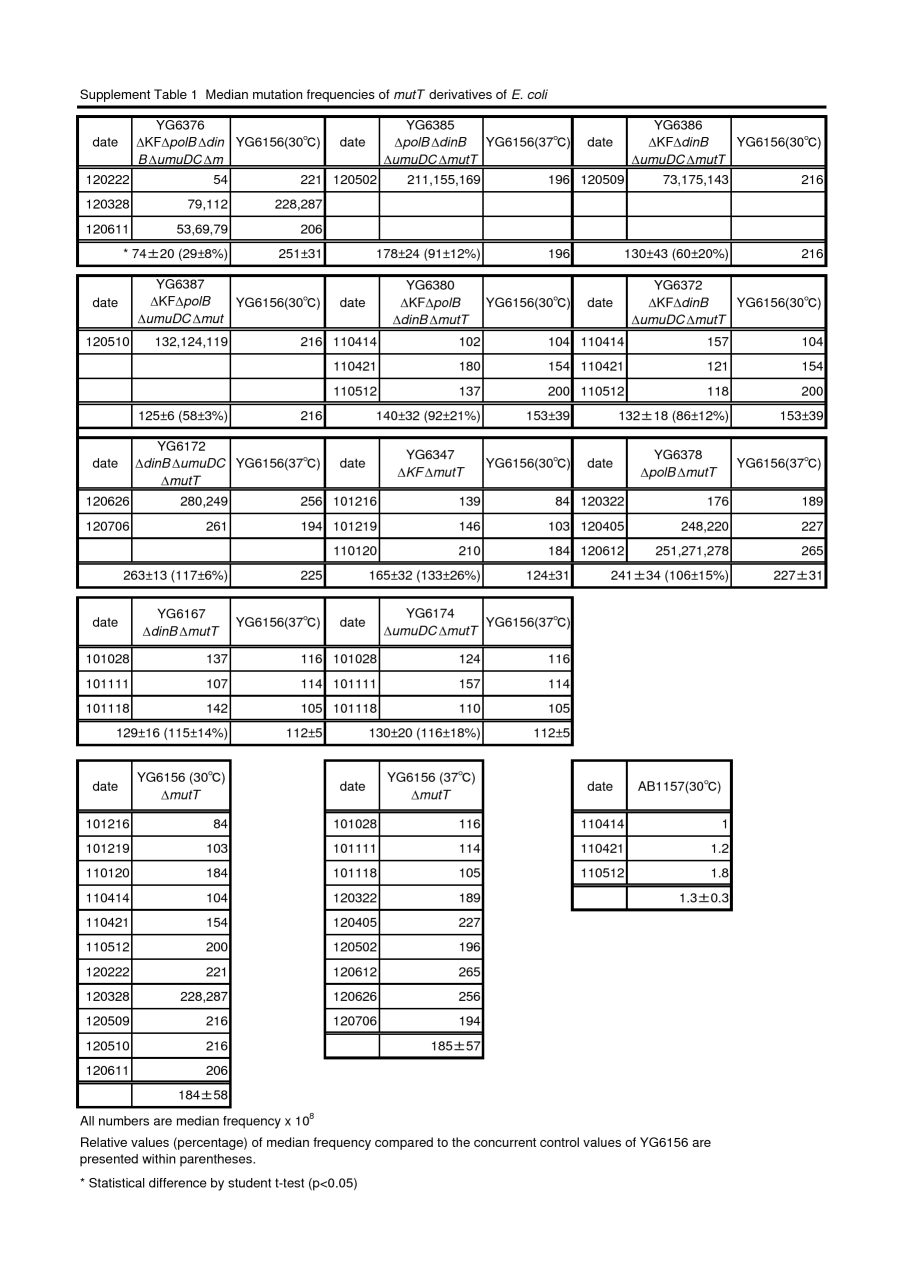

Supplement: Supplementary file 2 [file mmi0086-1364-SD2.png]
